# Development of a facile genetic transformation system for the Spanish elite rice paella genotype Bomba

**DOI:** 10.1007/s11248-022-00303-z

**Published:** 2022-04-13

**Authors:** Andrea Saba-Mayoral, Ludovic Bassie, Paul Christou, Teresa Capell

**Affiliations:** 1 Applied Plant Biotechnology Group, Department of Plant Production and Forestry Science, University of Lleida-Agrotecnio CERCA Center, Lleida, Spain; 2 ICREA, Catalan Institute for Research and Advanced Studies, Barcelona, Spain

**Keywords:** Direct DNA transfer, Genetic transformation, *Oryza sativa*, Zygosity determination

## Abstract

We report the development of an efficient and reproducible genetic transformation system for the recalcitrant Spanish elite rice paella genotype, Bomba. Preconditioned embryos derived from dry seeds were bombarded with gold particles carrying a plasmid containing a screenable and a selectable marker. We confirmed integration and expression of *hpt* and *gusA* in the rice genome. Transformation frequency was ca: 10% in several independent experiments. We show Mendelian inheritance of the input transgenes and zygosity determination of the transgenic lines in the T1 generation. A unique and critical step for the regeneration of plants from transformed tissue was shading during the early stages of regeneration, combined with a specific cytokinin:auxin ration at the onset of shifting callus to regeneration media.

**Supplementary Information:**

The online version contains supplementary material available at 10.1007/s11248-022-00303-z.

## Introduction

As societies evolved through history so have the plants early humans used for food and feed. The transition from gathering wild plants to cultivation involved gradual, increasing interactions between humans and the plants they used. The subsequent genetic changes in these plants resulted in the domestication and further development of cultivated species reflecting the ingenuity of early farmers, who were the first plant breeders (Vaughan et al. 2007). The current generation of plant breeders has tools available that enable them to engineer crops for specific applications and uses, ranging from agronomic and nutritional traits to specialty applications such as the production of recombinant pharmaceuticals and refined chemicals (Beyer 2010; Mehlo et al. 2005; Murad et al. 2020; Naqvi et al. 2009; Ramessar et al. 2008; Vamvaka et al. 2018; Wang et al. 2014; Zhu et al. 2008). This major step forward was catalyzed in part by the development of technology to introduce genes from any genetic source into plants. The resulting engineered crops exhibited dramatic increases in yield, robust resistance to biotic and abiotic stresses and novel characteristics, expanding their range of cultivation and use (Aragão et al. 2013; Ferreira et al. 2002; Fitch et al. 1992; Giron-Calva et al. 2020). The development of plant transformation methods involving *Agrobacterium tumefaciens* or physical methods such as direct DNA transfer, in combination with sophisticated cloning techniques permitted introduction of genes firstly into the nuclear plant genome, and subsequently into plastids (Altpeter et al. 2005; Majumder et al. 2021; Fuentes et al. 2018). The development of variety/genotype-independent transformation methods using immature embryos as explants permitted introduction of transgenes directly into elite germplasm in a single generation, eliminating the need for repetitive backcrossing (Christou et al. 1990, 1991; Kohli and Christou 2008).

More recently, multi-gene engineering permitted reconstruction and/or extension of biosynthetic pathways to generate plants with enhanced nutritional or pharmaceutical properties (Farre et al. 2014; Giuliano 2014; Vallarino et al. 2020; Zorrilla-Lopez et al. 2013). In the last decade, the arrival of genome editing (GEd) has resulted in another leap forward in crop breeding (Jansing et al. 2019; Ghogare et al. 2020). Importantly, GEd, in the case of knocking out endogenous genes leaves no other footprint in the genome and, at the sequence level, resulting indel mutants are indistinguishable from natural mutations or those induced by chemicals or radiation (Zhu et al. 2017).

Three billion people across the globe consume rice (*Oryza sativa* L.) daily and this grain makes up 20% of the world’s dietary energy supply (Birla et al. 2017). Rice is a strategic crop for food security as indicated by the Food and Agriculture Organization (FAO) due to its wider adaptability under diverse environmental conditions (Montano et al. 2014). Global rice consumption increased from 450 million tons in 2011 to ca: 490 million tons in 2020 and is projected to further increase to ca: 650 million tons by 2050 (Ristaino et al. 2021). Spain accounts for nearly 30% of rice production in the European Union. Rice cultivation in Spain is input-intensive and relies on certified seeds and major chemical inputs in the form of fertilizers, herbicides and pesticides. The reduced number of rice-specific pesticides as a result of more restrictive EU legislation represents one of the main challenges in the rice sector in European rice-producing countries in Southern Europe. Producers end up relying on the “exceptional authorization of pesticides” to deal with rice-specific pests and diseases (Gomez de Barreda et al. 2021). Rice in Spain is mainly cultivated in 6 of its 17 administrative regions and covers ca: 105,000 ha (Gomez de Barreda et al. 2021). Rice blast, caused by the filamentous ascomycete fungus *Magnaporthe oryzae*, is one of the most destructive diseases affecting rice in all rice-growing countries (including Spain) and often causes serious damage to global rice production (Dean et al. 2012; Liu et al. 2014). Weeds resistant to the most commonly used herbicides in rice cultivation in Spain (ALS and ACCase inhibitors), mainly in the areas of Extremadura and Marismas del Guadalquivir have been reported. Concerted efforts at raising awareness in the increase of weeds resistant to herbicides and the implementation of effective integrated weed management programs are needed (Juliano et al. 2010). Crop diversification and weed management practices, emphasizing non-chemical weed control tactics e.g. stale seedbed management before rice sowing (Chen et al. 2016) are important tools for the proactive management of herbicide-resistant weeds (Fisher et al. 2000; Kacan et al. 2020). Genetic engineering for the development of second and third generation herbicide tolerant elite rice varieties, in combination with the above agronomic practices, will give rice farmers additional and more effective options to manage their rice crop.

Early efforts to address as yet unmet challenges in Spanish rice improvement through genetic engineering started with the development of an Agrobacterium-based method for three elite genotypes using mature zygotic embryos as explants (Pons et al. 2000). We have used direct DNA transfer to develop an efficient and facile transformation system for Bomba rice, a variety preferred for its organoleptic and physical properties especially in the preparation of the typical Spanish dish “paella”. Bomba encompasses some of the oldest Spanish landraces which form a distinct and isolated cluster in a dendrogram using SNPs to trace relations, the origin and distribution of European rice cultivars (Reig-Valiente et al. 2016).

We characterized the transgenic plant population at the DNA and expression levels and report a straightforward method to determine homozygosity in T1 progeny. Our work sets the stage for the further genetic improvement of elite rice cultivars such as Bomba through conventional genetic engineering and/or genome editing.

## Experimental procedures

### Gene cloning, vector construction and rice transformation

We used plasmid pGUS-HPT which contains the pUC19 backbone (Yanisch-Perron et al. 1985) with the *β-glucuronidase* (*gusA*; Jefferson et al. 1987) and the *hygromycin B phosphotransferase* (*hpt*; Van den Elzen et al. 1985) genes, as screenable and selectable markers, respectively. The genes were under the control of separate 35S Cauliflower mosaic virus (CaMV) promoters and the *T-Nos* polyadenylation region as terminator (Christou et al. 1991). The 7619 bp sequence containing a single restriction site for *Xho*I at position 2171 bp and the 4972 bp *gusA-hpt* fragment was excised by the combination of *Xho*I and *Kpn*I restriction enzymes for subsequent molecular characterization of the transgenic lines (Supplementary Fig. S1A).

DNA-coated gold particles were prepared by mixing gold particles (10 mg; 1–3 μm in diameter) with a solution of the DNA (20 μg). Rice transformation was carried out as described in our previous publications (Valdez et al. 1998; Sudhakar et al. 1998). The device used for DNA delivery was a modified inflow gun which operates under a He pressure of 3100 kPa at a fixed distance of 2 cm between the orifice of the gun barrel and the target plate. These parameters result in transformation frequencies similar to those achieved by the standard BioRad He gun at a He pressure of 8200 kPa.

### Plant material

**Fig. 1:**
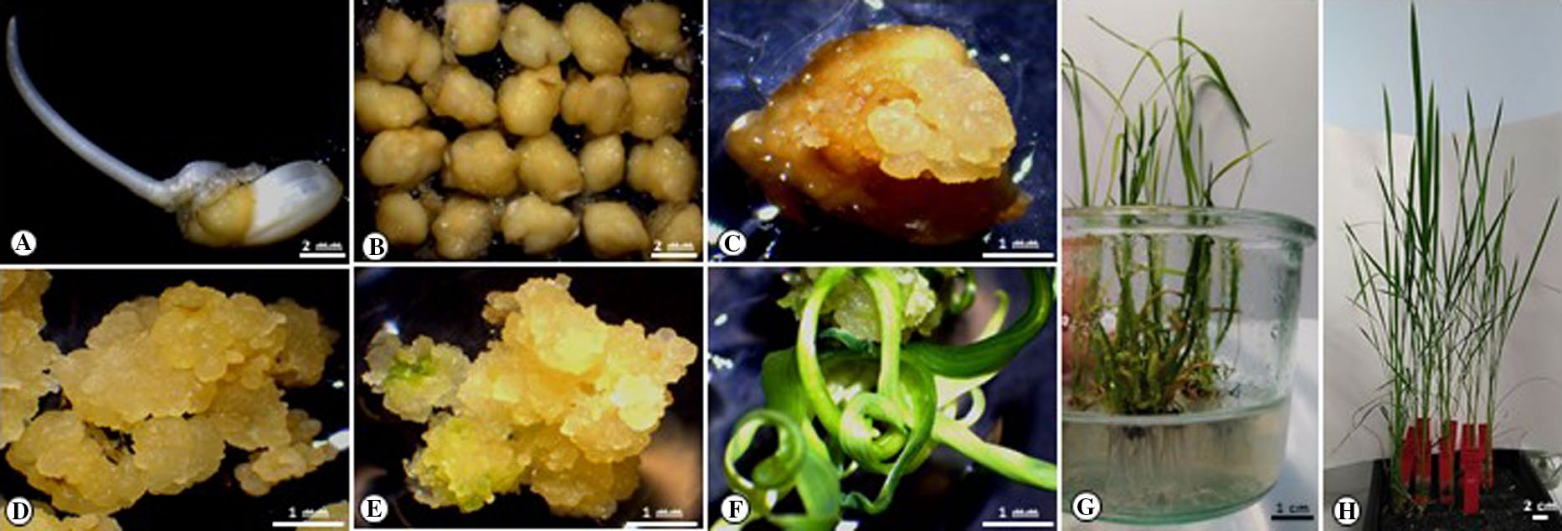
Bomba rice transformation system using mature seeds as explants. **A** Scutellum tissue 6 days after geminating seeds on proliferation medium; **B** seed-derived embryos (6 days after germination) immediately prior to bombardment; **C** hygromycin-resistant embryogenic callus on selection medium, 2 weeks after bombardment (prior to removal from the scutellum); **D** hygromycin-resistant embryogenic callus on selection medium 6 weeks after bombardment (after two successive subcultures of 2 weeks each); **E** regenerating (green) tissue derived from embryogenic callus after 2 weeks on regeneration medium under shade; **F** regenerating plantlet under selection; **G** plants on rooting medium; **H** established plants in soil

Rice seeds from *Oryza sativa*. *cv*. Bomba (provided by https://www.illaderiu.com/en/home-3/) were dehusked and surface-sterilized in 70% ethanol with continuous shaking for 5 min, placed in a 50% sodium hypochlorite solution with three drops of Tween for 30 min with agitation and rinsed five times in sterile distilled water. The seeds were dried on sterile filter paper and placed on Murashige-Skoog (Murashige and Skoog 1962) Proliferation Medium (4.4 g L^−1^ MSP including Gamborg B_5_ Vitamins, Duchefa Biochemie, Product No. M0231) supplemented with casein hydrolysate 300 mg L^−1^, proline 500 mg L^−1^, sucrose 30 g L^−1^, and 500 μL L^−1^ 2.4 D (from 5 mg mL^−1^ stock in ethanol), solidified with 3.5 g L^−1^ phytagel (0.35%), and incubated in the dark at 28 °C. The pH of the medium was adjusted to 5.8 using KOH before autoclaving at 120 °C for 20 min. Seeds were germinated for 5, 6 (Fig. 1A) or 7 days to assess time of embryo excision as an independent variable. Mature zygotic embryos were then separated from the endosperm and transferred to MS Osmoticum medium (MSO) (MS 4.4 g L^−1^ including Gamborg B_5_ Vitamins, casein hydrolysate 300 mg L^−1^, proline 500 mg L^−1^, sucrose 30 g L^−1^, and 500 μL L^−1^ 2.4 D (from 5 mg mL^−1^ stock in ethanol) and 72.8 g L^−1^ mannitol solidified with 3.5 g L^−1^ phytagel). Twenty mature embryos were put in each MSO petri dish for 4 h before and 16 h after bombardment (Fig. 1B). The mature embryo scutellum was orientated in such a way as to be in the direct path of the accelerated gold particles (Sudhakar et al. 1998; Valdez et al. 1998).

### Selection and plant regeneration

Twenty-four hours after bombardment, mature embryos were transferred to MSP and 2 days later to MSP medium supplemented with 30 or 50 mg L^−1^ hygromycin B (ref. 10843555001 Roche; MS Selection medium) and incubated in the dark at 28 °C for 2–3 weeks. Fast-growing embryogenic callus on the scutellum (Fig. 1C) was transferred to fresh MSS medium. After 6–8 weeks (with subcultures to fresh media every 2 weeks, Fig. 1D), hygromycin-resistant embryogenic callus was transferred to regeneration medium (MSR).

### Regeneration media

Four different regeneration media (different combinations of phytohormones) were used to improve the efficiency of plant recovery. MS basal medium (MS 4.4 g L^−1^ including Gamborg B_5_ Vitamins, casein hydrolysate 100 mg L^−1^, maltose 30 g L^−1^, 30 mg mL^−1^ hygromycin B and solidified with 3.5 g L^−1^ phytagel) was supplemented with 3 mg L^−1^ of 6 benzyl aminopurine (BA) or kinetin (Kin), in combination with 0.5 mg L^−1^ naphthalene acetic acid (NAA) or indoleacetic acid (IAA). The pH of all media was adjusted with KOH to 5.8 before autoclaving. Hygromycin and the phytohormones (BA, Kin, NAA and IAA) were filter-sterilized and added to the media after autoclaving. Embryogenic callus or green shoots were transferred to fresh media every 2 weeks, always maintaining the original hormone combination. Shading was highly beneficial at the early stages of regeneration (yellow light, mean irradiance 200 μmol m^−2^ s^−1^; Fig. 1E and F). Well-developed regenerating shoots were transferred to MSR Rooting Medium (MSRR; MS 2.2 g L^−1^ including Gamborg B_5_ Vitamins, sucrose 10 g L^−1^ and solidified with 3.5 g L^−1^ phytagel, Fig. 1G). Regenerated plants with a well-developed root system were transferred to soil in a growth chamber at 28/28 °C day/night temperature with a 12 h photoperiod (yellow light, mean irradiance 400 μmol m^−2^ s^−1^) and 80% relative humidity (Fig. 1H).

### Histochemical GUS assay

Histochemical GUS assays to monitor the transformation process were carried out using mature embryos (2 days after bombardment-transient expression, to assess effectiveness of DNA delivery) and in small segments of callus and leaves subsequently (to screen for potential stable transformants). Plant tissue was immersed in a GUS staining solution (100 mL L^−1^ sodium phosphate buffer (200 mM, pH = 7), 100 mg L^−1^ X-Gluc [Thermo Fisher Scientific™, Waltham, Massachusetts, USA) dissolved in 2 mL dimethyl sulfoxide, 0.2 mL L^−1^ Triton X-100, 4 mL L^−1^ EDTA (0.5 M, pH = 8), 42 mg L^−1^ K_3_Fe(CN)_6_)] and incubated at 37 °C for 4 h. The GUS staining solution was then removed and replaced with absolute ethanol to stop the enzymatic reaction (Jefferson et al. 1987; Christou 1992). Images of the embryos, callus and leaf segments were taken using a stereo-microscope (Moticam S3 camera and Motic Microscopy stereo zoom SMZ-171 BLED (Pole Type), Motic®, Hong Kong).

### Efficiency of DNA delivery and statistical analysis

**Fig. 2:**
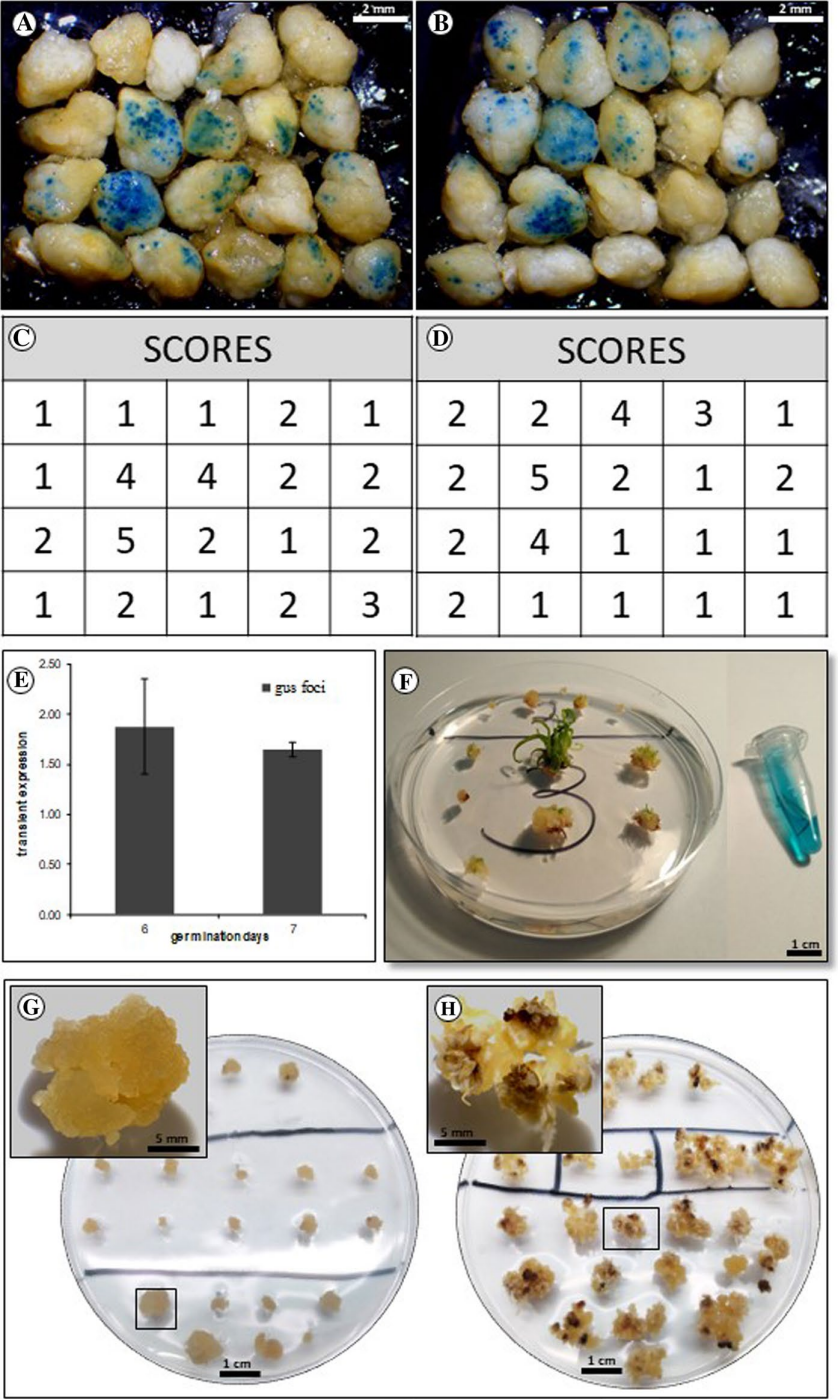
GUS expression in early transformation events on scutellum tissue. Transient GUS expression 48 h after bombardment: **A** 6 day-old and **B** 7 day-old embryos. **C**, **D** GUS scores based on the number of blue foci on scutellum tissue (from images **A** and **B**); **E** transient GUS expression measured 6 or 7 day-old embryos after bombardment (no significant difference at *P* ≥ 0.05); **F** plantlet regeneration after 3 subcultures on regeneration medium (with selection) and GUS expression in leaf; **G**, **H** impact of selection pressure (hygromycin at 30 mg L^−1^) on phenotype of regenerating embryogenic callus. Three week-old callus tissue on selection media (bombarded with pGUS-HPT); **G** inset: homogeneous embryogenic tissue; **H** inset: non embryogenic tissue (bombarded only with gold particles-no transgenic DNA)

The relative quantification of GUS-expressing foci on the scutellum after bombardment was done by giving five different scores (1–5) depending on the number of blue foci on the scutellum as shown in Fig. 2A–E. The score of blue foci of the 20 embryos was used as the final value per plate. Statistical analysis was done using the final value of all replicates per treatment. Results are given as mean (n = 6) ± standard error (SE). Analysis of variance by a *t*-Test using the residual mean square in the ANNOVA as the estimate of variability was performed.

### DNA extraction

DNA was isolated following the procedure of Creissen and Mullineaux (1995). Tissue samples (callus and leaf, 300 mg) were ground in a porcelain mortar using liquid nitrogen. The powder was added to a 2 mL Eppendorf tube containing 750 µL of extraction buffer (500 mM NaCl, 100 mM Tris–HCl pH 8 and 50 mM EDTA pH 8) and 75 µL 20% (w/v) SDS. The samples were mixed by vortexing for 10 min and incubated at 65 °C for 1 h. One volume of phenol–chloroform-isoamyl alcohol (25:24:1) was then added and samples were centrifuged (5 min at ca: 13,000×*g*). Supernatant was removed and 6 μL RNAase (10 mg mL^−1^) was added and incubated 30 min at 37 °C. Phenol extraction was repeated one more time. The supernatant was removed and added to an equal volume of isopropanol to precipitate the DNA. After centrifugation (10 min at ca: 13,000×*g*), the DNA pellet was washed with 1 mL 70% ethanol and suspended in autoclaved millipore water (100 μL). DNA was quantified using a Nanodrop 2000c spectrophotometer (Thermo Fisher Scientific, Waltham, MA, USA).

### PCR analysis

Genomic PCR amplification was carried out in a total volume of 20 µL, containing 2 µL DreamTaq™ Green Buffer 10 × with 20 mM MgCl_2_ (Thermo Fisher Scientific™, Waltham, Massachusetts, USA), 0.2 µM dNTP (10 µM; Thermo Fisher Scientific™), 0.2 µM of forward and reverse primers (10 µM), 0.125 µL DreamTaq DNA Polymerase (5U μL^−1^ Thermo Fisher Scientific™, Waltham, Massachusetts, USA), and 1 µL of DNA (1000 ng µL^−1^) and autoclaved millipore water. For the PCR reaction DNA was denatured at 96 °C for 2 min followed by 30 amplification cycles (denaturation at 92 °C for 1 min, annealing at 60 °C for 1 min and extension at 72 °C for 1 min) and kept at 72 °C for 10 min. The *hpt*-coding sequence was detected by using: forward primer Hpt-1-F 5′-ACTCACCGCGACGTCTGTCG-3′ and reverse primer Hpt-1-R 5′-GATCTCCAATCTGCGGGATC-3′ (Supplementary Fig. S1B). The predicted amplification product size was 1271 bp. PCR products were resolved using agarose gel (1%) electrophoresis 1 × Tris–acetate buffer (TAE). The transforming plasmid was used as positive control and DNA from wild-type (wt) plants as negative control.

### DNA blot hybridization

Twenty micrograms of genomic DNA (callus and leaf tissue), extracted as described above, were digested with *Xho*I (which cuts once at position 2171 bp in the transforming plasmid) or with the combination of *Xho*I-*Kpn*I (which releases the 4972 bp cassette with the *gusA* and *hpt* coding region; supplementary Fig. S1A) as recommended by the supplier (Thermoscientific FastDigest, Lithuania). Serial dilutions of genome-equivalent amounts of the digested plasmid pGUS-HPT containing the transgene of interest were used as a template to generate the positive controls for 1, 3 and 6 copies. For example, 380 pg of pGUS-HPT (Supplementary Fig. S1B) was calculated to be equivalent to 1 copy of 20 µg of the rice genome (400 Mb; Sasaki and Burr 2000). To represent 3 and 6 copies, 1140 pg and 2280 pg were used, respectively. Digested genomic DNA was separated on a 0.8% TAE agarose gel as described in Capell et al. (2004). DNA was transferred to a positively charged nylon membrane (Roche, UK). Nucleic acids were fixed by UV crosslinking at 120 m Joules for 2 min 30 s. Membranes were washed in 2xSSC for 30 min twice, and then pre-hybridized at 42 °C for 2 h using the DIG-easy hybridization solution (Roche, UK). The 466 bp *hpt* and 676 bp *gusA* probes were labeled with the DIG-system using the PCR DIG probe synthesis kit (Roche, UK). Alkali-labile DIG-11-dUTP was incorporated into the probe as follows: PCR was set up in a final volume of 50 μL containing 400 μM dATP, dCTP and dGTP; 320 μM dTTP, 80 μM DIG-11-dUTP, 10 × PCR buffer (50 mM KCl, 10 mM Tris–HCl pH 9.0, 0.1% Triton X-100); 2.5 U of Taq DNA polymerase (Roche, UK), 0.1 μM of both forward and reverse sequence primers (Stab vida, Caparica, Portugal) and 500 pg of the transforming plasmid as template. After an initial denaturation step of 2 min at 96 °C, 30 cycles of PCR were performed using a thermal cycler. Each cycle involved denaturation at 92 °C for 1 min, annealing at 56 °C for *hpt* and 64 °C for *gusA*, for 1 min and strand extension at 72 °C for 1 min. Last step was 72 °C for 10 min. The forward sequence primer (5'-ACTCACCGCGACGTCTGTCG-3') started from position 15 in the *hpt* cDNA open reading frame. The reverse primer sequence was 5'-ATACACATGGGGATCAGCAATC-3'. The resulting amplification product was 466 bp. The forward sequence primer (5′-ACGGCAAAGTGTGGGTCAATAATC-3′) started from position 281 bp in the *gusA* cDNA open reading frame and the reverse primer sequence was 5′-TCCATTAATGCGTGGTCGTGCACC-3′. The amplification product was 676 bp. The two PCR-labeled probes were purified using the QIAquick Gel Extraction Kit (Qiagen UK) and denatured at 95 °C for 10 min prior to use. Hybridization was performed at 42 °C overnight. The membranes were washed twice for 5 min in 2xSSC, 0.1% w/v SDS at room temperature, twice (20 min) in 0.5xSSC, 0.1% w/v SDS at 68 °C, once (15 min) 0.2xSSC, 0.1% SDS at 68 °C, and then once (15 min) 0.1xSSC, 0.1% SDS, at 68 °C. Chemiluminescence detection was performed according to the manufacturer's instructions using DIG Luminescent Detection Kit (Roche, UK). After washing, the membranes were incubated with CSPD® Chemiluminescent Substrate (Roche, UK) for 30 min at 37 °C and subsequently exposed to BIORAD Molecular Imager® ChemiDoc™ XRS^+^ for 20 min at RT. Stripping and reprobing the membranes with the *gusA* gene was performed as described in Hloch et al. (2001).

### RNA extraction and cDNA synthesis

Total RNA was obtained from rice callus by phenol:chloroform:isoamyl alcohol (25:24:1) extraction and LiCl (4 M) precipitation (Creissen and Moulineaux 1995). The RNA was quantified using a Nanodrop 2000c spectrophotometer (Thermo Fisher Scientific, Waltham, MA, USA), and 1 μg of total RNA was used as the template for first-strand cDNA synthesis with Maxima™ H minus cDNA Synthesis Master Mix with dsDNase kit (Thermo Fisher Scientific, Waltham, MA, USA) in a 20 μL total reaction volume according to the manufacturer’s recommendations.

### Gene expression analysis by real-time quantitative PCR

Real-time Quantitative PCR (RT-qPCR) was performed in 96-well plates on a CFX96 system (Bio-Rad) using a 15 µL mixture containing, 10 ng cDNA template, 1× iTaq™ Universal SYBR® Green Supermix (Bio-Rad) and 0.25 µM of each primer; reactions were performed in triplicate. Amplification primer-specificity was confirmed by melting curve analysis of the final PCR products in the temperature range 50–90 °C, and 62 °C was chosen as the optimal temperature for all primers. To calculate primer efficiency, five serial dilutions (from 5 to 25-fold diluted cDNA) were used to produce standard curves for each gene. The efficiency of the *hpt*, *gusA* and actin primers were within the accepted limits to permit comparison across genes in terms of mRNA expression levels (McElroy et al. 1990; Xiujie et al. 2019). To calculate relative expression levels, serial dilutions (0.2–125 ng) were used to produce standard curves for each gene. Triplicate PCRs in 96-well optical reaction plates were carried out with the following profile: a heating step of 3 min at 95 °C was followed by 40 cycles at 95 °C for 10 s, 62 °C for 30 s (at the optimal primer temperature), and 72 °C for 20 s. *Hpt* and *gusA* mRNA expression were normalized vs actin using the 2^−ΔΔ*C*t^ method (Livak and Schmitten 2001). The fluorescence threshold value and gene expression data were calculated using CFX96 system software. Primer combinations are listed in Supplementary Fig. 1B.

### Stability and inheritance of introduced transgenes

Stability of the transgenes integrated into the rice genome was performed by DNA blot analysis. DNA extraction, blot analyses and calculation of copy number genome-equivalents were performed as described above. Twenty micrograms of genomic DNA from T_0_ and 10 T_1_ progeny plants were digested with *Xho*I and probed with the *hpt* and *gusA* probes. Homozygosity was confirmed by analyzing 30 T_2_ progeny from each of 10 T_1_ plants by PCR as described above.

## Results

### Critical parameters for the development of an efficient transformation system for Bomba rice

**Fig. 3:**
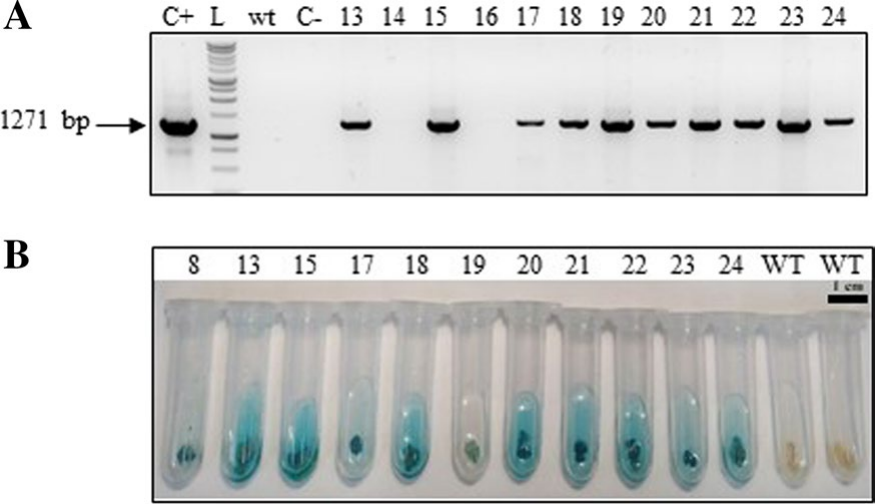
DNA and GUS analyses. **A** PCR from genomic DNA of callus lines transformed with pGUS-HPT. Lane C +  contains the PCR product from pGUS-HPT used for bombardment, as a positive control. Lane L contains the 1 kb DNA Ladder (ThermoFisher Scientific SM0313). Lane wt contains the PCR product from a wilt type (wt) plant. Lane C- contains the master mix used in the PCR reaction without DNA. Lanes 13 to 24 contain the PCR products amplified from independent callus lines. Samples 14 and 16 were negative. The expected PCR product for the *hpt* gene is 1271 bp; **B** Gus expression analysis of different callus lines and wt

We tested 3 different callus induction times (5, 6 and 7 days; 6 days shown in Fig. 1A and B) to assess efficiency of DNA delivery. Isolated mature embryos were transformed by particle bombardment using a plasmid carrying the *hpt* selectable marker that encodes for resistance to hygromycin B and the *gusA* screenable marker gene (Supplementary Fig. 1A). The scutellar region of the embryo was bombarded as described (Sudhakar et al. 1998; Valdez et al. 1998). Bombarded tissues (Fig. 1B) were plated on selection media supplemented with hygromycin B at different levels (ranging from 20 to 50 mg L^−1^). Embryogenic callus appeared 10–12 days following bombardment (Fig. 1C). No significant differences were observed in the efficiency of callus induction between embryos bombarded at 6 or 7 days on proliferation media (*P* ≥ 0.05; Table 1 and Fig. 2A, B and E). No callus developed on embryos bombarded after 5 days, possibly because longer induction times are required for target cells to become competent for regeneration and transformation, similarly to earlier observations in soybean using a completely different regeneration system (Christou and Yang 1989; Christou and McCabe 1992). Embryos selected at 30 mg L^−1^ hygromycin produced two distinct callus phenotypes and these were subsequently correlated directly with transgenic plant recovery (Fig. 2G and H). Selection at hygromycin levels below 30 mg L^−1^ was ineffective, whereas no callus proliferated on any embryos selected at hygromycin levels higher than 30 mg L^−1^. Most proliferating callus (ca: 70%; Fig. 2G) was compact and embryogenic. However, a slower-growing non-embryogenic callus also developed and proliferated (Fig. 2H), even after successive rounds of selection at 30 mg L^−1^ hygromycin. Selected embryogenic callus was transferred every 2 weeks to fresh selection media but this did not eliminate completely the non-embryogenic callus. Tissues bombarded with naked gold particles (no DNA) exclusively produced non-embryogenic callus (at ca: 30% frequency; Fig. 2H) which was able to grow on media supplemented with hygromycin (30 mg L^−1^), even after successive sub-cultures. Hygromycin at 50 mg L^−1^ completely inhibited growth of callus under every condition we tested, irrespectively of whether the callus was transformed or not. Therefore, selection pressure alone is not sufficient to distinguish between transformed and non-transformed tissues in the Bomba genotype. However, an embryogenic phenotype selected visually upon subsequent subcultures on media containing hygromycin (30 mg L^−1^) assured very high transformation frequencies and no escapes. On average, over ca: 90% of embryogenic callus (Table 1) was subsequently shown to be transformed by molecular analyses (Fig. 3A) and regenerated transgenic plants. We were thus able to distinguish between selection efficiency and overall transformation efficiency (Table 1). We defined selection efficiency as the % of independent callus lines exhibiting an embryogenic phenotype over surviving callus (irrespective of phenotype). Transformation efficiency was defined as the % of confirmed transformed lines (at the DNA level) over the number of bombarded embryos. A selection efficiency of ca: 90% and a transformation frequency of ca: 8–10% were obtained from embryos isolated after 6 or 7 days on proliferation media, respectively, indicating no significant differences due to the days of incubation on proliferation media (*P* ≥ 0.05; Fig. 2E and Table 1). *GusA* transient expression was observed 24 h post transformation (Fig. 2A and B).

**Table 1:** Selection and transformation efficiencies

Induction time	Number of explants	Explants with embryogenic callus	Number of transformed callus	Selection efficiency (%)	Transformation efficiency (%)
6 days	180	14	13	92.86	7.22
	300	34	28	82.35	9.33
	100	11	10	90.91	10
Average				88.7	8.85
7 days	380	46	41	89.13	10.79
	80	6	6	100	7.5
	200	30	25	83.33	12.5
Average				90.82	10.26

### A specific hormone combination is essential for efficient plant regeneration from transformed Bomba callus

After 6–8 weeks on selection media (with sub-culture to fresh media every 2 weeks) embryogenic callus selected under a microscope was transferred to regeneration media (MSR) and kept under a white piece of filter paper for the first 2 weeks. MSR medium was supplemented with phytohormones (BA, Kin, NAA and IAA) and 30 mg L^−1^ hygromycin B. Selection pressure was only removed for rooting. Supplementary Table 1 shows the number of green shoots recovered per Petri plate for each hormone combination. A combination of BA (3 mg L^−1^)/NAA (0.5 mg L^−1^) resulted in the highest regeneration frequency defined as the number of green shoots obtained per Petri plate (52%; Supplementary Table 1). Fully developed plants were recovered after 3 rounds of subculture on MSR (Fig. 2F). Putative transgenic plants were screen for GUS prior to in depth molecular characterization (Fig. 2F).

### DNA integration analysis and transgene copy number determination

Genomic DNA was analysed by PCR to amplify a 1271 bp fragment of the *hpt* cDNA. In total, 120 callus lines proliferating on selection media from 6 independent transformation experiments were screened by PCR. One hundred lines were generated from callus recovered after transformation with pGUS-HPT and 20 callus lines from control experiments using only gold for bombardment (no DNA). Tissues were also tested for *gusA* expression. Ninety-six of the 100 putative transgenic lines (Fig. 3A) showed GUS expression (96%) after 3 rounds of subculture on MSS (Fig. 3B). All plantlets regenerated from these lines, also expressed GUS (Fig. 2F). Our analysis revealed that ca: 96% of the lines we analyzed co-expressed the two transgenes. Figure 3 shows representative results from a number of independent lines.

**Fig. 4:**
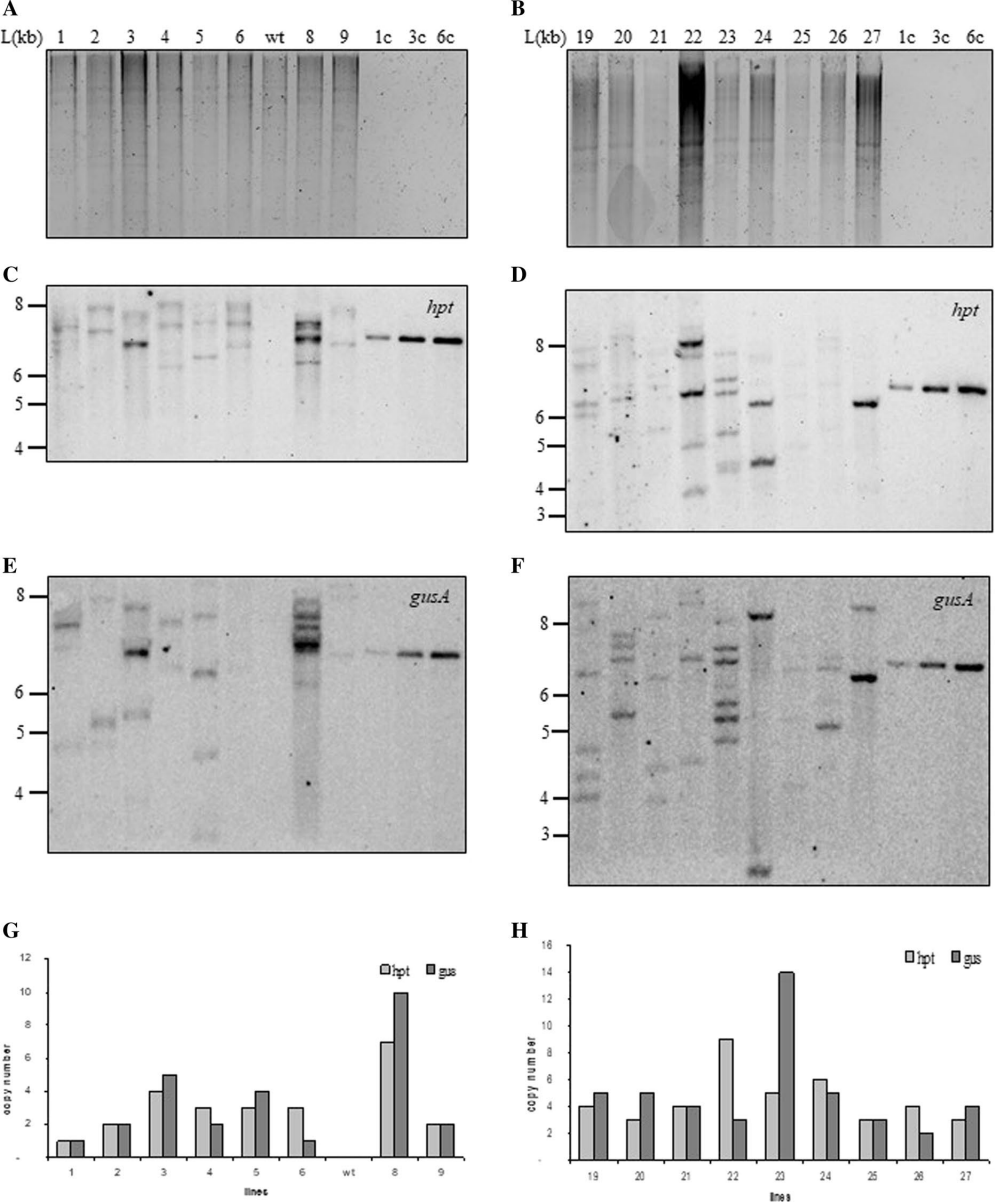
Gel blot analyses of *Xho*I-digested genomic DNA (20 μg) from independent lines. **A**, **B** Ethidium bromide-stained gel showing complete digestion of genomic DNA; **C**, **D** blot probed with the 466-bp-DIG-labelled PCR product from *hpt*; **E**, **F** blot probed with the 676-bp-DIG-labelled PCR product from *gusA*; **G**, **H** copy number of each line calculated from comparisons with pGUS-HPT genome-equivalent amounts. L, 1 kb DNA Ladder (ThermoFisher Scientific SM0313) in kilobases (kb). Numbers indicated independent transformed lines; wt, wild type; 1, 3 and 6 copies of *Xho*I digested pGUS-HPT

Genomic DNA was digested with *Xho*I (Fig. 4A and B) which cuts once in the transforming plasmid backbone upstream of the CaMV 35S promoter (Supplementary Fig. 1) and hybridized with the 466 bp *hpt* (Fig. 4C and D) and the 676 bp *gusA* (Fig. 4E and F) DIG-labeled PCR products. This confirmed integration of the selectable (*hpt*) and the screnable marker genes in all 20 transgenic lines we tested and confirmed their independent origin (Fig. 4 shows 17 of these lines; 3 remaining lines were run on a different membrane-results not shown). Transgene copy number was estimated by counting the number of the *hpt* and *gusA* hybridization bands and comparing the intensity of bands with the linearized 7619 bp positive control pGUS-HPT (1, 3 and 6 copies; Fig. 4). We defined “insertion” as the physical presence of a transformation cassette in the genome. Each insertion may comprise different copies of the transgene as observed by comparing with the intensity of the standards (for 1, 3 and 6 copies of the plasmid) (Fig. 4). We classified transgenic lines in three categories: low copy number (3 or less copies: 50% for *hpt* and 45% for *gusA*), mid copy number lines (between 4 and 6 copies: 35% for *hpt* and 40% for *gusA*) and high copy number lines (7 or more copies: 15% for both genes; Fig. 4G and H). Comparing the total of *hpt* versus *gusA* insertions in the same line, in 70% of the lines the number of integrated copies as well as the integration patterns of *hpt* and *gusA* were different (Fig. 4).

**Fig. 5:**
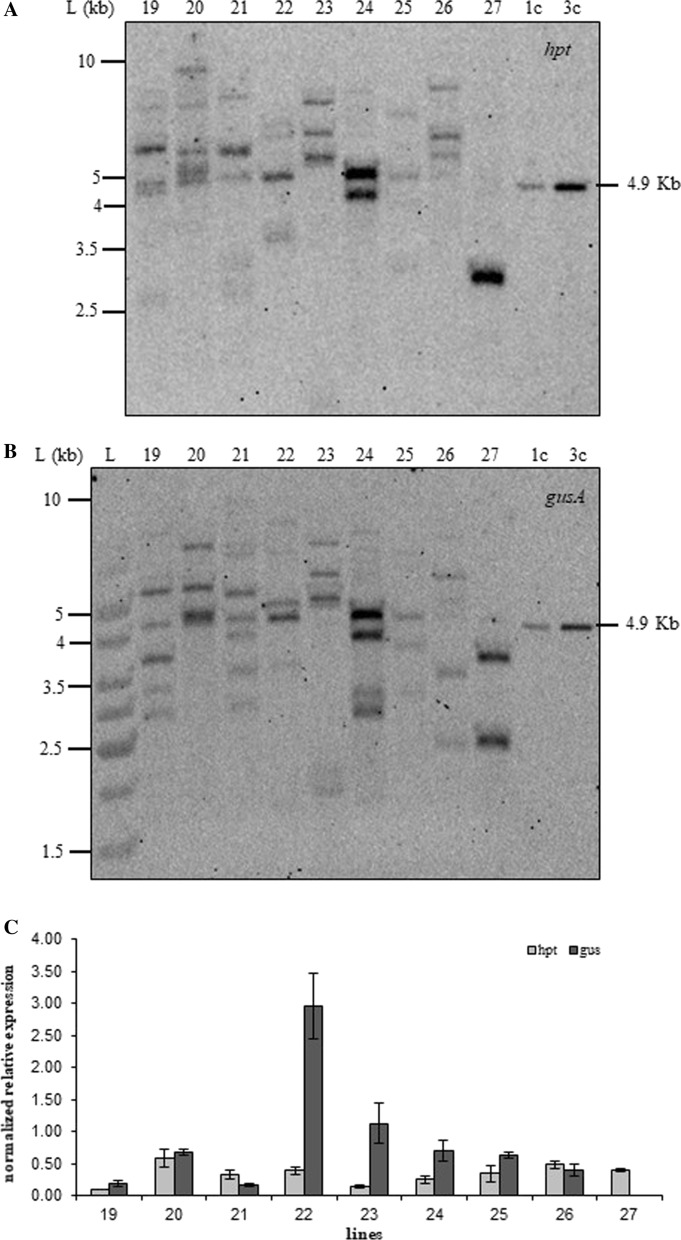
Gel blot analysis of *Xho*I-*Kpn*I-digested genomic DNA (20 μg) from independent lines and gene expression analyses. **A** Blot probed with the 466-bp-DIG-labelled PCR product from *hpt*; **B** blot probed with the 676-bp-DIG-labelled PCR product from *gusA*. L, 1 kb DNA Ladder (ThermoFisher Scientific SM0313) in kilobases (kb). Numbers indicate independent transgenic lines; wt, wild type; 1, and 3 copies of *Xho*I-*Kpn*I digested pGUS-HPT. **C** Normalised relative mRNA expression for the *hpt* and *gusA* genes

DNA blot hybridization of genomic DNA digested with the combination *Xho*I–*Kpn*I (double cut in pGUS-HPT releasing a 4972 bp fragment) with the *hpt* and *gusA* probes showed that 70% of the lines contained the full length cassette cDNA for both *hpt* and *gusA* (for example lines 19, 20 and 21; Fig. 5A and B, respectively). The remaining lines had higher or lower size bands in relation to the full length cassette cDNA (Fig. 5A and B; representative lines 23, 26 and 27). When comparing *hpt* and *gusA* integration patterns, we also identify a different banding pattern in all lines (Fig. 5A and B).

### Transgene expression

We selected thirty independent transformants, representing a molecular diversity (different transgene copy numbers) observed in 100 callus lines to determine if any correlations could be made between transgene copy number and expression of the selected *hpt* and the non selected *gusA* genes. All transformed lines expressed *hpt* at the mRNA level, as expected. *GusA* expression was detected in 96% of the lines analyzed. Significantly higher levels of mRNA *hpt* expression over *gusA* were measured in 17% of the lines (2 to threefold higher; Fig. 5C); whereas *gusA* mRNA expression was significantly higher over the *hpt* gene in 47% of the lines (2 to ninefold higher; Fig. 5C). No significant differences in the levels of expression between the two genes were measured in the remaining 36% of the lines (*P* ≥ 0.05; Fig. 5C). Plants that did not show *gusA* expression did not have the full cassette cDNA for the *gusA* gene as expected (Fig. 5B and C).

### Homozygosity determination of transgenic T1 plant lines

**Fig. 6:**
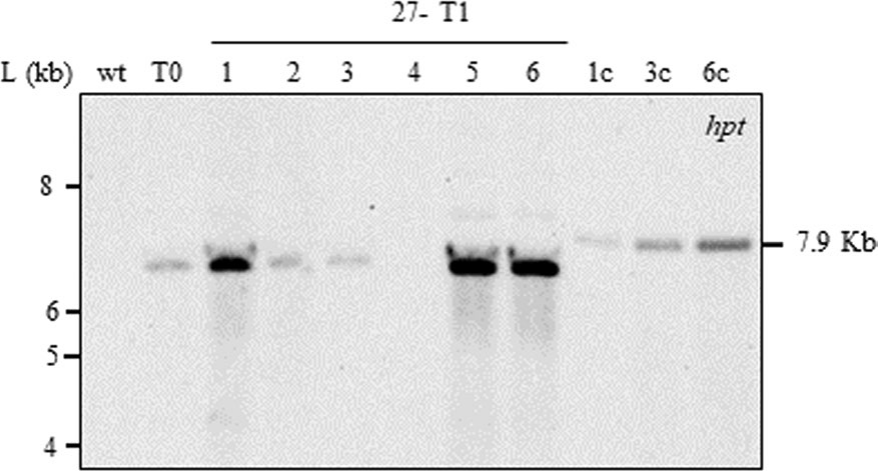
Gel blot analysis of *Xho*I-digested genomic DNA (20 μg) (progeny plants). Blot probed with the 466-bp-DIG-labelled PCR product from *hpt*; L, 1 kb DNA Ladder (ThermoFisher Scientific SM0313) in kilobases (kb). wt, wild type; T0 plant #27; numbers indicate T1 progeny plants; 1, 3 and 6 copies of *Xho*I digested pGUS-HPT

Ten progeny plants (six shown in Fig. 6) from transgenic plant line 27 were analysed for the presence of the transgene by DNA blot analysis. Genomic DNA digestion with *Xho*I and probing the membrane with the *hpt* showed that the transgene segregated in the progeny consistently with single locus insertion (Mendelian inheritance 3:1). When compared T1 intensities to the T0 parent, we estimated that plants 27–1, 27–5 and 27–6 were homozygous (Fig. 6). PCR analyses for the *hpt* gene of 30 T_2_ progeny plants from plants 27–1, 27–5 and 27–6 showed presence of the transgene in all plants confirming homozygosity of the line.

## Discussion

Rice genetic transformation has come a long way since the early reports of the recovery of transgenic plants from immature embryo explants (Christou et al. 1991). Even though the system is genotype independent (for elite japonica and indica varieties) reliance on the use of immature embryos is a constraint as such explants may not be available at all times. Subsequent transformation systems utilizing mature seed-derived embryos provided more flexibility but they are not as broadly applicable to all genotypes and locally adapted varieties (Valdez et al. 1998; Sudhakar et al. 1998). Even though rice transformation is viewed as routine, being able to transform local varieties is far from routine, particularly if mature seed-derived embryos are used as explants. Transformation of three Spanish commercial varieties, Senia, Tebre and Bahia using mature seed-derived embryos has been reported (Pons et al. 2000). Successful transformation was achieved after a detailed optimization of parameters involved in the *Agrobacterium* transformation and the regeneration process (Pons et al. 2000).

We report the development of an efficiency transformation system for the elite Spanish variety Bomba using mature seed-derived embryos (Fig. 1). We encountered and resolved a number of unanticipated challenges in the process. Unlike other plant genotypes (including but not limited to rice) the Bomba genotype was shown to be very sensitive to selection pressure. When the level of hygromycin exceeded a certain threshold in the culture medium, callus did not proliferate at all from the transformed explants. Hygromycin selection pressure of 30 mg L^−1^ resulted in the recovery of a mixture of embryogenic and non-embryogenic callus (Fig. 2G and H) whereas hygromycin levels below 30 mg L^−1^ were completely ineffective. Both callus types were able to proliferate under selection (30 mg L^−1^) but only the embryogenic callus regenerated efficiently to transgenic plants (Fig. 3A and B). Thus transformation of Bomba not only requires optimal selection pressure but equally importantly requires the recognition of an embryogenic callus phenotype (under selection at 30 mg L^−1^) which can be propagated and subsequently regenerated to transgenic plants at high frequency (ca: 90%). When the selection step in the transformation of any plant species is not very efficient, as in the case of the Bomba genotype, careful visual selection and propagation of highly embryogenic callus from a piece of proliferating callus with mixed phenotypes is extremely important and in our view the key to the successful recovery of transgenic plants at high frequency. Visarada et al. (2002) described the effect of induction media on the morphology of embryogenic callus in different rice genotypes. They also described the importance of visual selection of embryogenic callus to increase the efficiency of plant regeneration. Many elite and locally adapted rice varieties cannot be transformed effectively because selection is inefficient.

After 2 weeks of transferring the embryogenic tissue to regeneration media with partial shading, green tissue began to form. The hormone combination of BA (3 mg L^−1^)/NAA (0.5 mg L^−1^) gave the highest frequency of regeneration (Supplementary Table 1) and was chosen for the subsequent tissue culture steps.

Transgenic callus lines were transformed with a construct that contained two genes, *hpt* and *gusA*. DNA-gel blot analysis demonstrated that 20 randomly chosen independent lines contained both transgenes. We observed a range of 1 to 10 copies of each transgene per haploid genome (Fig. 4). Even though the two genes were on the same plasmid the number of integrated transgenes was different in most of the lines indicating that the plasmid undergoes rearrangements prior or during integration into the genome. Kohli et al. (1999) characterized and identified that rearrangements may occur in the coding region or in the promoter of the gene. In earlier reports sequencing of novel recombination junctions suggested that the transforming plasmid had undergone rearrangements involving illegitimate recombination in monocots (Altpeter and Xu 2000; Gahakwa et al. 2000; Varshney and Altpeter 2001) as well as in dicots (Romano et al. 2003a, 2003b). Kohli et al. (1999) also established that specific properties of the CaMV 35S promoter resulted in a cluster of recombination events in the promoter region confirming the predominance of microhomology-mediated recombination. Since the same construct was used in our transformation experiments (as the one used in Kohli et al. 1999) the complex DNA integration patterns we observed were most likely caused by fragmentation of the input plasmid and recombination before integration. For example, lines 24 and 27 showed similar copy number for *hpt* and *gusA* (mid copy number); however, when probed with the *hpt* and *gusA* genes the banding pattern was very different (Fig. 4D and F) indicating clear rearrangements of the input plasmid into the rice genome.

When the entire expression cassette was released with the aim of evaluating its integrity into the rice genome, we observed a different integration pattern for the *hpt* and *gusA* genes (Fig. 5). Probing the membrane with the *hpt* gene, washing and subsequent reprobing with the *gusA* gene confirmed the rearrangements. However, the presence of the full length cassette for both genes coincided in the same lines (Fig. 5A and B) indicating that the restriction sites were intact and rearrangements took place outside the coding regions. All 14 lines (out of the 20 lines we analyzed) contained the full intact expression cassette (for both transgenes). In four of the remaining lines which had higher size bands, *hpt* and *gusA* bands coincided. A similar situation was observed in the remaining 2 lines which had lower size bands than expected (Fig. 5A and B).

Earlier literature reported that transgene copy number could be positively or negatively correlated to levels of expression (Hobbs et al. 1993). In our experiments we show that transgene expression was not influenced by copy number similarly to earlier reports (Altpeter et al. 2005; Naqvi et al. 2010). Higher expression levels were detected in plants with multiple copies of *hpt* and *gusA*. For example, line 22 contained 9 and 3 copies of *hpt* and *gusA*, respectively (Fig. 4D and F). The corresponding relative mRNA expression of *hpt* and *gusA* were 0.39 ± 0.06 and 2.95 ± 0.50 (measured as 2-ΔCq ± SE). These levels were 2 and 24 –fold higher when compared to expression of the two transgenes in line 9 (containing two copies of each transgene) with relative mRNA levels of 0.19 ± 0.012 and 0.12 ± 0.008 (measured as 2-ΔCq ± SE) for *hpt* and *gusA*, respectively. The different levels of expression of the transgenes can be attributed to a number of factors, other than copy number, including fragmented or rearranged transgene copies, position effects, among others. High and stable levels of expression of introduced transgenes rely on the formation of a SMART locus, i.e. one containing Stable Multiple Arrays of Transgenes (Naqvi et al. 2010). Thus, a transgenic locus containing multiple genes or multiple gene copies, which is stable through meiosis so as to avoid segregation, rearrangement or epigenetic silencing will assure maximum and stable expression of introduced transgenes.

In conclusion, we report the development of a highly efficient transformation system for the elite Spanish rice genotype Bomba. Our system will now permit the development of novel Bomba rice varieties to address current challenges in Spanish rice cultivation and production through genetic engineering or genome editing. These include resistant to major pests and diseases, enhanced nutrition and value-added products, among others.

## Supplementary Information

Below is the link to the electronic supplementary material.

**Supplementary table legend: Table 1** Plantlet regeneration frequency (number of plantlets per Petri dish) on MSR media supplemented with different hormone combinations. Each Petri plate contained 20 callus pieces from the same line. Supplementary file1 (XLSX 9 kb)

**Supplementary figure legend: Figure 1**. Schematic representation of plasmid pGUS-HPT used in transformation and primers used. (A) Map of pGUS-HPT vector. The *hygromycin phosphotransferase* (*hpt*) and *β -glucuronidase* (*gusA*) transcriptional units are driven by the 35S Cauliflower mosaic virus promoter (CaMV 35S). Both genes have the *T-Nos*, *A. tumefaciens* nopaline synthase terminator. *Xho*I was used to linearize the plasmid. *Kpn*I/*Xho*I was used to release the full length *hpt-gusA* expression cassette. (B) Primer pairs for PCR and probes. Supplementary file2 (JPG 57 kb) Supplementary file2 (JPG 56 kb)
