# Supplementary figures and images for: Development of a facile genetic transformation system for the Spanish elite rice paella genotype Bomba
Source: Transgenic Res. 2022 Apr 13;31(3):325–40. doi: 10.1007/s11248-022-00303-z (PMC9135871; doi:10.1007/s11248-022-00303-z)

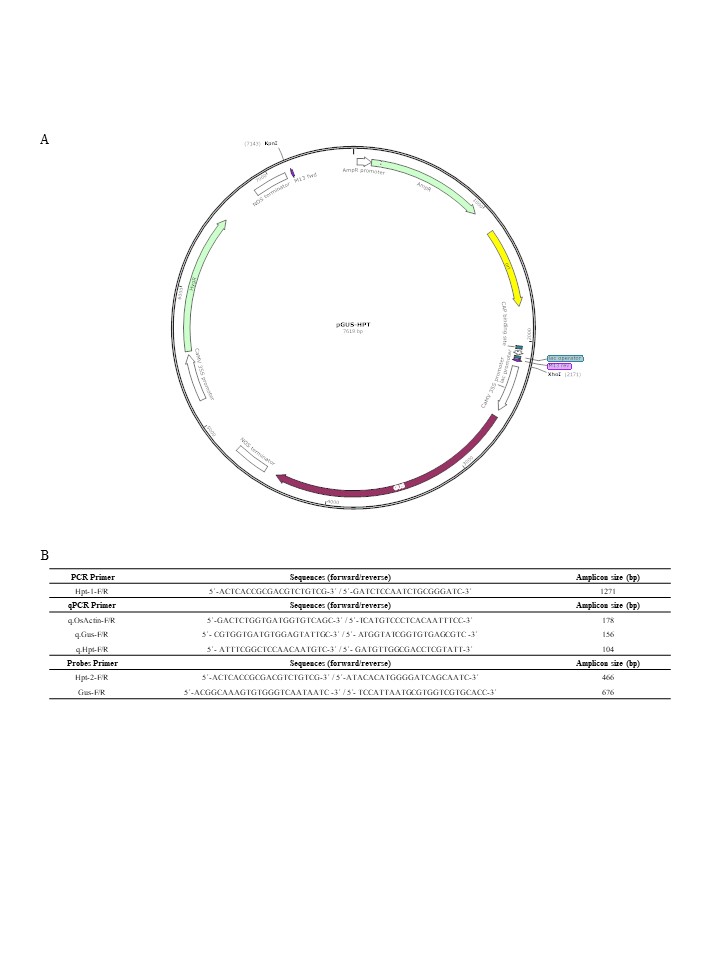

Supplement: Supplementary file 2 — Supplementary figure legend: Figure 1. Schematic representation of plasmid pGUS-HPT used in transformation and primers used. (A) Map of pGUS-HPT vector. The hygromycin phosphotransferase (hpt) and β -glucuronidase (gusA) transcriptional units are driven by the 35S Cauliflower mosaic virus promoter (CaMV 35S). Both genes have the T-Nos, A. tumefaciens nopaline synthase terminator. XhoI was used to linearize the plasmid. KpnI/XhoI was used to release the full length hpt-gusA expression cassette. (B) Primer pairs for PCR and probes. Supplementary file2 (JPG 57 kb) [file 11248_2022_303_MOESM2_ESM.jpg]
